# A novel inverse association between cord 25-hydroxyvitamin D and leg length in boys up to three years. An Odense Child Cohort study

**DOI:** 10.1371/journal.pone.0198724

**Published:** 2018-06-11

**Authors:** Mathilde Egelund Christensen, Signe Sparre Beck-Nielsen, Christine Dalgård, Søs Dragsbæk Larsen, Sine Lykkedegn, Henriette Boye Kyhl, Steffen Husby, Henrik Thybo Christesen

**Affiliations:** 1 Department of Clinical Research, Faculty of Health Sciences, University of Southern Denmark, Odense, Denmark; 2 Hans Christian Andersen Children’s Hospital, Odense University Hospital, Odense, Denmark; 3 Department of Pediatrics, Kolding Hospital a part of Lillebaelt Hospital, Kolding, Denmark; 4 Department of Regional Health Research, Faculty of Health Sciences, University of Southern Denmark, Odense, Denmark; 5 Department of Public Health, Environmental Medicine, University of Southern Denmark, Odense, Denmark; 6 Odense Child Cohort, Hans Christian Andersen Children’s Hospital, Odense University Hospital, Odense, Denmark; 7 Odense Patient data Explorative Network (OPEN), University of Southern Denmark, Odense, Denmark; Universidade de Sao Paulo, BRAZIL

## Abstract

**Background and aim:**

Long standing vitamin D deficiency in children causes rickets with growth impairment. We investigated whether sub-ischial leg length (SLL) is shorter, and cephalo-caudal length:length (CCL:L) ratio and sitting height:height (SH:H) ratio larger, with lower cord s-25-hydroxyvitamin D (25OHD) in the population-based prospective Odense Child Cohort, Denmark.

**Methods:**

We included healthy singletons born to term with available measures of cord 25OHD and anthropometrics up to three years’ age. Linear regression was stratified by sex *a priori* and adjusted for maternal ethnicity, pre-pregnancy body mass index and smoking during pregnancy, season of blood sampling and child age.

**Results:**

Median (IQR) cord 25OHD was 48.0 (34.0–62.4) nmol/L. At mean age 19.1 months, n = 504, mean (SD) SLL was 31.7 (1.7) cm; CCL:L-ratio 0.62 (0.01). At 36.3 months, n = 956, mean SLL was 42.9 (2.0) cm; SH:H-ratio 0.56 (0.01). No participants had rickets. In adjusted analyses, 19-months-old boys had 0.1 cm shorter SLL (p = 0.009) and 0.1% higher CCL:L-ratio (p = 0.04) with every 10 nmol/L increase in cord 25OHD. Similar findings were seen for late pregnancy 25OHD. In the highest cord 25OHD quartile (>60.7 nmol/L), SLL was 0.8 cm shorter (95% C.I.: 1.36;-0.29, linear trend, p = 0.004), and CCL:L-ratio 0.8% higher (95% C.I. 8.0x10^-05^;0.01, linear trend, p = 0.01), compared to lowest quartile (<30.7 nmol/L). Similar associations with cord 25OHD were observed in 3-year-old boys. No consistent associations between 25OHD and anthropometrics were seen in girls at either age.

**Conclusion:**

No leg shortening was found with decreasing cord s-25OHD in a healthy population of infants. A small, yet significant inverse association between cord 25OHD and SLL in boys 1½-3 years warrants further investigations.

## Introduction

Vitamin D is well known for its calciotropic effects being pivotal to bone mineralization and linear bone growth in children. Severe vitamin D deficiency in children manifests clinically as rickets characterized by mineralization failure of the growth plates and of the newly formed osteoid at the bone matrix [1]. Rickets exerts a broad palette of symptoms including disturbed apoptosis of the chondrocytes leading to cartilage hyperplasia of the growth plates in the metaphyseal bone with widening of the growth zones at wrists, ankles and knees [2]. Furthermore, long bone growth impairment and bowing of weight-bearing long bones may lead to disproportional linear growth with shortening of the sub-ischial leg length (SLL) and thereby an increased ratio of sitting height to height (SH:H). Hypovitaminosis D may be defined as vitamin D deficiency or insufficiency (serum-25-hydroxyvitamin D (25OHD) < 25 nmol/L and 25–50 nmol/L, respectively) [3], although the most appropriate cut points are still under debate [4, 5]. The existing cut-off limits are generated in terms of adult bone-health and the applicability to children remains unknown [4–8]. In pregnancy, an increase in the active vitamin D calcitriol (1,25(OH)_2_D) ensures calcium and phosphate supply to the foetus. In foetal life, parathyroid-hormone-related protein (PTHrP) is the primary regulator of the mineral homeostasis and bone mineralization, which in the foetus is independent of maternal vitamin D and occurs uncomplicated as long as sufficient calcium and phosphorous is supplied from the maternal circulation across the placenta. This transport is independent of active vitamin D [9, 10]. Maternal 25OHD crosses the placenta and, as the foetus has no endogenous synthesis of 25OHD, maternal vitamin D insufficiency during pregnancy implies vitamin D insufficiency in the new born. This leaves the infant more susceptible to developing vitamin D deficiency rickets if not receiving adequate vitamin D supplementation, as the lower inborn vitamin D stores will decrease to severely insufficient levels sooner in accordance with the half-life of 25OHD.

Inborn rickets due to vitamin D deficiency, however, is very rare, but may be seen in children of mothers with severe vitamin D deficiency, osteomalacia and low s-calcium during pregnancy [11]. Cord 25OHD levels only amount to a fraction of the levels in the maternal circulation at time of delivery [12, 13], in part caused by placental production of the enzyme, CYP24A1, responsible for the biodegradation of 25OHD and 1,25(OH)_2_D_3_ [14–16]. Low foetal vitamin D stores may therefore further decrease to critical levels soon after birth, in the absence of infant vitamin D supplementation.

Skeletal manifestations of vitamin D deficiency rickets rarely becomes apparent after weeks, more often after months from birth [10]. A Danish study found vitamin D deficiency rickets to be diagnosed between 0.3 to 3.6 years of age with a median age at diagnosis of 1.4 years [17]. Although maternal vitamin D status during pregnancy has no impact on foetal bone mineralisation if calcium supply is adequate, observational studies have linked low maternal vitamin D levels with smaller child bone mass and impaired linear growth in the first 12 months of life [18–20]. Results are conflicting regarding linear growth in older children [21–23].

Vitamin D deficiency and insufficiency are still common in pregnancy and neonates [24]. In the Municipality of Odense, Denmark, s-25OHD <50 nmol/L was observed in 27.8% of early pregnant women, in 15.8% of late pregnant women [25], but in 57.7% of the cord blood samples [26]. If low maternal or cord 25OHD is linked to diminished infant growth, especially of the long bones, despite vitamin D supplementation recommendations, an important potential for enforced prevention strategies is suggested, even in populations with low incidence of vitamin D deficiency rickets.

On this background we aimed to investigate the relationship between early life vitamin D exposure and linear growth in children up to three years of age in the large, Danish, population-based Odense Child Cohort (OCC).

## Methods

**Fig 1:**
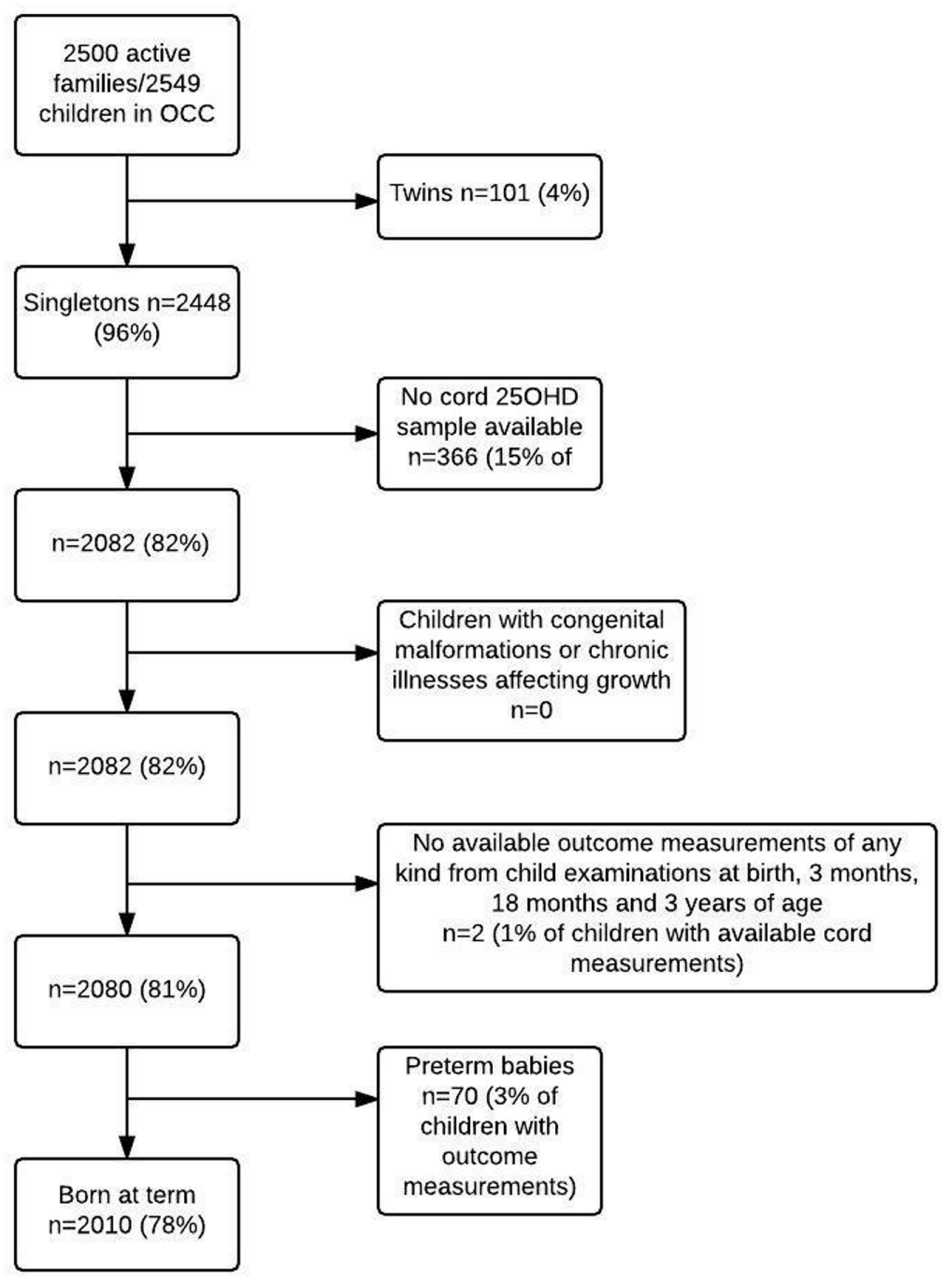
Flowchart of inclusion of 2010 participants from the Odense Child Cohort. Including healthy singletons born at term. Abbreviations: 25OHD: 25-hydroxyvitamin D.

OCC is an on-going prospective birth cohort recruiting pregnant women residing in the Municipality of Odense, Denmark, between January 1^st^ 2010 and December 31^st^ 2012 as earlier described in details [27]. The present analyses included 2080 women and their singletons with available cord 25OHD analyses and anthropometric measures of the child at three months, 18 months and three years of age. We excluded preterm infants (born before gestational week 37+0) and singletons with severe congenital malformations or severe chronic diseases affecting growth (Fig 1).

### Assessment of 25OHD

Blood samples from early and late pregnancy (mean gestational age (GA) 12 and 29 weeks, respectively) and mixed arterial/venous blood from cord were analysed for serum 25OHD concentration by liquid chromatography tandem mass spectrometry (LC-MS/MS) using a Thermo Scientific TLX1 system attached to a Thermo Scientific Vantage TSQ. The limit of quantification was 0.15 nmol/L for both 25OHD_2_ and 25OHD_3_, but only concentrations above 6.5 nmol/L were considered. Vitamin D status was reported as the sum of 25OHD_2_ and 25OHD_3_ concentrations [25]. All serum samples were stored at -80°C until analysis. The analysis was externally calibrated against National Institute of Standards and Technology, Standard Reference Material 972 [28].

### Leg length, body ratios and length/height

Birth length was extracted from hospital files. Data from OCC follow-up visits at age three months, 18 months and three years were extracted for total length/height, cephalo-caudal length (CCL) at 18 months and sitting height (SH) at three years. CCL and SH were subtracted from total length/height to generate the SLL measure.

CCL and total length were measured once to the nearest mm in supine position using a SECA 216 infantometer in children younger than two years of age. Height was measured once in standing position using a SECA 213 stadiometer to the nearest mm in children from two years. SH was calculated as the mean of three measurements to the nearest mm using a SECA 213 stadiometer with the child sitting on a standard sitting chair of known height.

### Covariates

We obtained data on maternal height, pre-gestational weight and pre-gestational body mass index (BMI) from the general practitioner’s files. Maternal age, smoking habits, parity and GA at delivery were retrieved from hospital files and infants were considered preterm when GA<37+0 weeks. Maternal skin-tone (Fitzpatrick-scale, where type I/II or type V/VI), and paternal height were extracted from self-reported questionnaire data obtained prior to the first antenatal visit to the OCC. Ethnicity and maternal educational level was provided by the Municipality and ethnicity was defined as western or non-western according to parental origin. Women were categorized as smokers if they reported smoking during early pregnancy regardless of quantity. Time spent outdoors during pregnancy and vitamin D supplementation during pregnancy was self-reported in a questionnaire at 28 weeks of gestation. Gestational weight gain was self-reported in a questionnaire approximately three months after birth. At child age 1½ year, duration of exclusive breastfeeding (weeks) and intake of infant formula after eight weeks of age (yes/no) were reported. Infant diet at this time was registered as number of dairy/fish/meat servings of any kind consumed on a daily basis with no regard of the type of product being ingested. Dietary intake of calcium and vitamin D intake were estimated by multiplying the frequency of self-reported consumed food with standard portion sizes [29] and information obtained from Danish food composition tables [30] The intake of supplementary vitamin D was calculated based on self-reported data on type and frequency of supplements, and a database of all registered supplements in Denmark provided by the Danish Veterinary and Food Administration [31]. Birth season was defined as winter (December-February), spring (March-May), summer (June-August) and autumn (September-November) representing seasonal fluctuations in 25OHD concentrations [25].

### Statistical methods

We used visual inspection of QQ-plots to estimate data distributions, presenting mean and standard deviation (SD) for normally distributed data and median and interquartile range [IQR] for skewed data. When comparing the means of two groups, unpaired two-sided t-tests were used for normally distributed data and Mann-Whitney for skewed data.

Age- and gender-adjusted Z-scores were generated for length/height and birth length for term infants in monthly intervals using updated Danish reference curves by Tinggaard *et al*. [32]. Values exceeding three SD from the reference mean were considered data entry errors and were excluded from further analyses (n = 5).

Cord 25OHD was chosen as primary exposure and SLL at 1½ year as primary outcome. Crude associations between continuous values of 25OHD and all outcomes were examined using linear regression. The associations among boys (*n* = 281) and girls (*n* = 239) were evaluated separately.

We modelled the adjusted associations between pregnancy and cord 25OHD and all anthropometric outcomes using ordinary linear regression. Measures of 25OHD were used in the model as either a continuous variable or as a categorical variable grouped by quartiles and linear trends were examined using the Wald test. To control for confounding, the following variables were included in all models: pre-gestational BMI, maternal ethnicity, smoking during pregnancy, season of birth, and child age at examination. These covariates were selected among all the variables defined in the section above through stepwise exclusion from the linear model if they did not change the beta coefficient estimate by more than 10% in the primary association between cord 25OHD and SLL at 1½ year. The variables in the above were chosen in accordance with the current literature.

QQ-plots of residuals were used to evaluate model assumptions for linear regression models. No violations were found. Data analysis was performed using STATA 14.0 (StataCorp). Significance level was defined as p<0.05 using two-sided tests.

### Ethics

The study fulfilled the Helsinki II declaration. The Regional Committees on Health Research Ethics for Southern Denmark (S-20090130) and the Danish Data Protection Board (case number 13/14088) approved the OCC and the present study. The parents of the participating children gave written informed consent based on verbal and written information.

## Results

Of the 2549 OCC participants, we included 2010 (79%) children who were born at term and had available cord 25OHD samples and anthropometrics. Of these, 504 (21%) children (231 girls, 273 boys) had their SLL measured at the second follow-up visit at a mean (SD) age of 19.1 (0.9) months. SLL was measured in 956 children (39%; 455 girls, 501 boys) at the third follow-up at a mean age of 36.3 (0.8) months. *Post hoc* power calculation indicated an 80% probability of detecting an association between cord 25OHD and SLL, if SLL changed a minimum of 0.09 cm with every 10 nmol/Lchange in cord 25OHD.

Characteristics of the 2010 infant-mother pairs included are presented according to 25OHD quartiles, Table 1. The median cord 25OHD concentration was 45.2 [30.7–60.7] nmol/L, with 17% < 25 nmol/L and 40% in the insufficient range 25–50 nmol/L. Compared to children in the highest cord 25OHD quartile, children in the lowest quartile were more often born during winter and spring and more likely had mothers of non-western ethnicity, mothers with pre-gestational BMI above 25 kg/m^2^, mothers with parity above three children, mothers who took less than the recommended vitamin D supplement of 10 μg/day, or mothers who smoked during pregnancy, Table 1.

**Table 1:** Basic characteristics of the 2010 participants.

	N	Quartiles of cord 25OHD, nmol/L				p-value*
		< 30.7	30.8–45.1	45.2–60.6	> 60.7	
**Maternal characteristics**						
Maternal height, cm	2010	168.3 ± 6.5^1^	168.0 ± 6.6	168.2 ± 6.1	168.5 ± 6.1	0.7
Maternal pre-gestational weight, kg	2010	69 [60–80] ^2^	66 [60–74]	66 [59–75]	65 [59–73]	**<0.001**
Pregestational BMI, kg/m^2^	2010	24.3 [21.8–28.1]	23.3 [21.4–26.0]	23.5 [21.1–26.2]	22.8 [21.0–25.6]	**<0.001**
Gestational weight gain, kg	873	15.1 ± 6.3	15.0 ± 5.6	15.5 ± 5.6	14.5 ± 5.2	0.4
Maternal age, y	2010	29.8 ± 4.6	30.4 ± 4.3	30.3 ± 4.3	30.0 ± 4.6	0.1
Smoking in pregnancy, *n* (%)						**<0.001**
Smoker	98	43 (8.6)	18 (3.6)	19 (3.8)	18 (3.6)	
Non-smoker	1912	460 (91.4)	484 (96.4)	484 (96.2)	484 (96.4)	
Maternal educational level, *n* (%)						0.2
Lower	252	74 (19.5)	64 (16.3)	54 (13.7)	60 (17.7)	
Intermediate	908	218 (57.5)	230 (58.7)	245 (62.2)	215 (63.2)	
Higher	345	87 (23.0)	98 (25.0)	95 (24.1)	65 (19.1)	
Ethnicity, *n* (%)						**<0.001**
Western	1918	459 (93.2)	482 (95.8)	486 (98.6)	491 (97.1)	
Non-western	92	44 (6.8)	20 (4.2)	17 (1.4)	11 (2.9)	
Skin-type**, *n* (%)						0.2
I/II White	299	86 (22.6)	84 (21.3)	66 (16.7)	63 (18.4)	
III Darker white	889	207 (54.3)	239 (60.5)	249 (62.8)	194 (56.7)	
IV Brown	309	82 (21.5)	67 (16.9)	78 (19.7)	82 (23.9)	
V/VI Dark brown	17	6 (1.6)	5 (1.3)	3 (0.6)	3 (1.0)	
Parity, *n* (%)						**<0.001**
First child	1109	230 (45.7)	282 (56.2)	295 (58.7)	302 (60.2)	
Second child	679	204 (40.6)	164 (32.7)	156 (31.0)	155 (30.9)	
Third child or more	222	69 (13.7)	56 (11.1)	52 (10.3)	45 (8.9)	
Time spent outdoors, mother, *n* (%)						**0.002**
Never/rarely	31	11 (2.9)	7 (1.8)	7 (1.8)	6 (1.8)	
Sometimes	295	95 (24.9)	84 (21.2)	62 (15.7)	54 (15.8)	
Often	920	204 (54.4)	252 (63.6)	256 (64.7)	208 (60.8)	
Most of the time	270	72 (18.8)	53 (13.4)	71 (17.8)	74 (21.6)	
Vitamin D supplementation in pregnancy, mother, *n* (%)						**<0.001**
≤10 μg/day	157	64 (23.4)	33 (10.7)	36 (11.4)	24 (8.5)	
>10 μg/day	1023	210 (76.6)	275 (89.3)	281 (88.6)	257 (91.5)	
**Paternal characteristics**						
Paternal height, cm	1223	182.6 ± 6.7	182.5 ± 7.0	183.2± 6.6	182.3± 6.7	0.3
**Child characteristics**						
Gestational age (GA) at birth, w	2010	40 [39–41]	40 [39–41]	40 [39–41]	40 [39–41]	0.6
Season of birth *n* (%)						**<0.001**
Winter (Dec-Feb)	489	190 (37.7)	143 (28.5)	107 (21.3)	49 (9.8)	
Spring (Mar-May)	496	158 (31.4)	144 (28.7)	106 (21.1)	88 (17.5)	
Summer (Jun-Aug)	477	29 (5.8)	78 (15.5)	135 (26.8)	235 (46.8)	
Autumn (Sep-Nov)	548	126 (25.1)	137 (27.3)	155 (30.8)	130 (25.9)	
Child sex, *n* (%)						0.6
Girls	948	241 (47.9)	228 (45.4)	247 (49.1)	232 (46.2)	
Boys	1062	262 (52.1)	274 (54.6)	256 (50.9)	270 (53.8)	
Child age at 18 months examination, m	1350	18.7 [18.3–19.4]	18.8 [18.2–19.4]	18.0 [18.4–19.6]	19.0 [18.4–19.5]	**0.03**
Duration of exclusive breastfeeding, w	1230	18 [6–34]	19 [11–24]	20 [10–24]	20 [12–24]	0.06
Intake of infant formula after 8 weeks of age						0.06
Yes	92	19 (6.5)	21 (6.2)	34 (10.3)	18 (5.2)	
No	1219	275 (93.5)	319 (93.8)	297 (89.7)	328 (94.8)	
Dietary intake of calcium, mg/day	1243	489 [213–756]	495 [230–751]	495 [224–757]	489 [211–747]	0.06
Dietary intake of vitamin D, μg/day	1185	1.14 ± 1.01	1.18 ± 1.66	1.04 ± 0.90	0.98 ± 1.0	0.2
Vitamin D supplementation, μg/day	943	3.47 ± 2.21	3.98 ± 1.36	3.88 ± 1.45	4.03 ± 1.59	**0.002**

ANOVA was used for comparing means of numerical, normally distributed variables. Kruskall-Wallis was used for comparing medians of numerical variables not normally distributed. Chi squared was used for comparing distributions of categorical variables. Abbreviations: 25OHD: 25-hydroxyvitamin D.

^1^ Median [IQR] (all such numbers).

^2^ Mean ± SD (all such numbers).

*p-value for difference between lowest and upper cord 25OHD quartile.

**Skin-type (I-IV) refers to Fitzpatrick scale.

The mean (SD) length/height was 52.0 (2.3) cm at birth, 63.2 (2.8) cm at three months; 84.0 (3.0) cm at 19 months, and 96.8 (3.6) cm at three years, S1 Table.

At 19 months, the mean SLL was 32.0 (1.48) cm for boys, 31.3 (1.78) for girls; CCL:L-ratio 0.62 (0.01) for each of the both sexes. At three years, SLL was 43.1 (1.98) cm for boys, 42.8 (2.00) cm for girls and SH:H-ratio was 0.56 (0.01) for boys, 0.55 (0.01) for girls. No children were diagnosed with rickets in the study period.

### Long bone length and cord 25OHD

**Fig 2:**
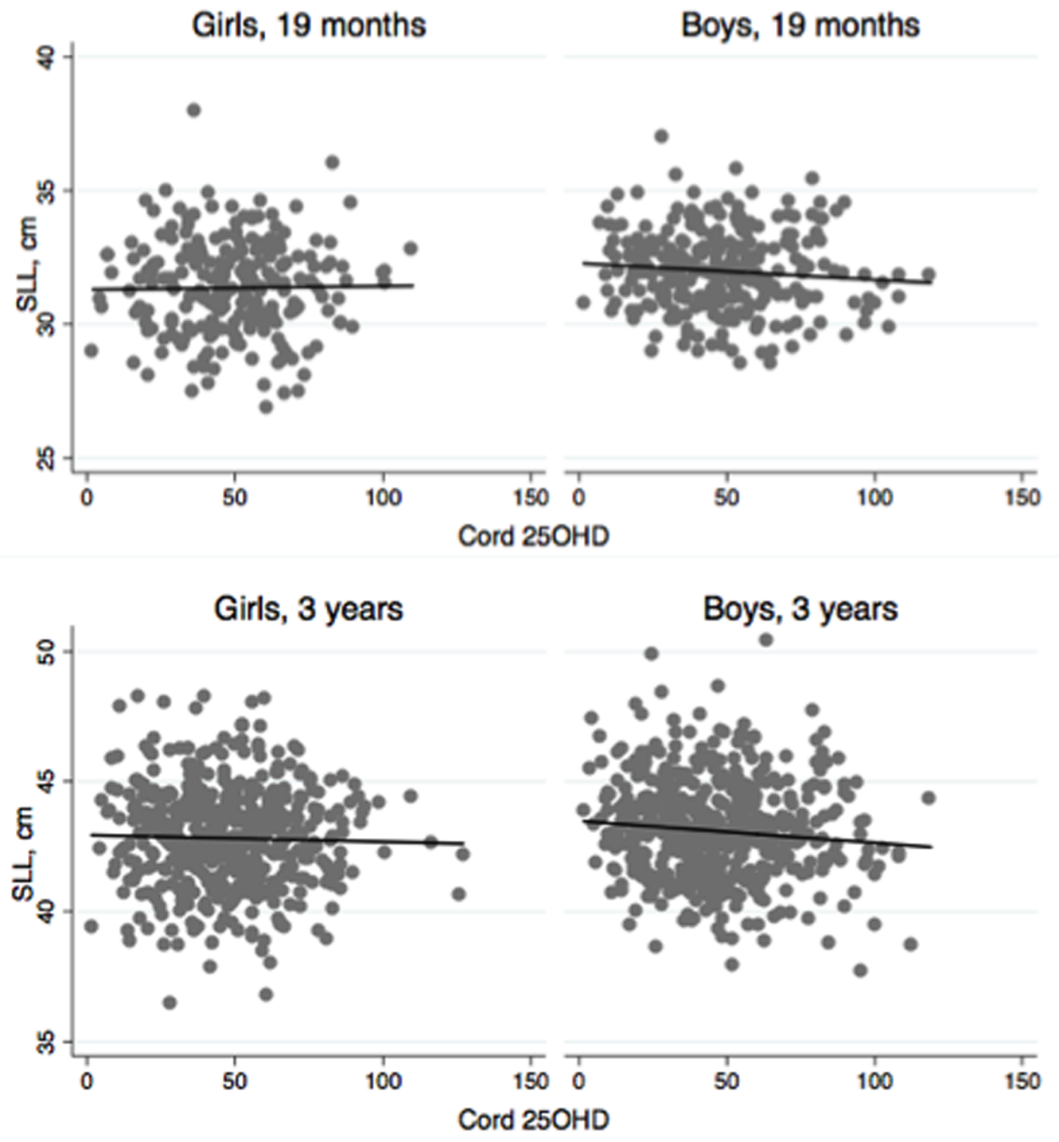
Scatterplot of the crude association between cord 25OHD and SLL at 19 months and three years by child sex. Sub-ischial leg lengths (SLL) in cm for cord 25-hydroxyvitamin D (25OHD) in nmol/L fitted with a linear model. No significant, crude associations were observed between cord 25OHD and SLL in either sex. Abbreviations: SLL: sub-ischial leg length. 25OHD: 25-hydroxyvitamin D.

We observed no crude associations between continuous values of cord 25OHD and our primary outcome, SLL at 19 months, Fig 2 and S2 Table (girls p = 0.8, boys p = 0.2). To our surprise however, SLL for boys at three years was inversely related to cord 25OHD (p = 0.03). No such crude association was seen in girls (p = 0.5). No crude associations were observed between cord 25OHD and total length at 3-months-old boys, 19 months or three years of age.

In the adjusted linear model, cord 25OHD was inversely associated with SLL in19 months-old boys, SLL decreasing 0.1 cm for each 10 nmol/L increase in cord 25OHD, p = 0.001, Table 2. Moreover, SLL was 0.8 cm shorter for boys with cord 25OHD levels >60.7 nmol/L (Q4) compared with boys with levels <30.7 nmol/L (Q1), with a dose-response relationship across cord 25OHD quartiles (linear trend p = 0.004), Table 2, In line with these observations, the CCL:L-ratio in 19-months-old boys was 0.0008 higher for every 10 nmol/L increase in cord 25OHD (p = 0.04) and boys with cord 25OHD in Q4 had a 0.006 higher CCL:L ratio compared to boys with levels in Q1 (linear trend, p = 0.01), Table 3.

**Table 2:** Adjusted associations between cord S-25-hydroxyvitamin D and leg length at 19 months and three years for girls and boys. The linear regression was adjusted for maternal pre-gestational BMI, smoking in pregnancy, maternal ethnicity, season of birth and exact child age at examination. Stratified by sex *a priori*.

	Girls			Boys		
	N	β (95% CI)	p	N	β (95% CI)	p
**SLL, 19 months**						
25OHD	231	-0.003 (-0.02;0.01)	0.6	273	-0.01 (-0.02;-0.003)	**0.009**
25OHD quartiles						
ref: Q1						
Q2	52	-0.20 (-0.93;0.53)	0.6	67	-0.30 (-0.83;0.23)	0.2
Q3	71	-0.13 (-0.82;0.57)	0.7	76	-0.46 (-1.00;-0.07)	**0.04**
Q4	65	-0.33 (-1.06;0.40)	0.4	72	-0.82 (-1.38;-0.26)	**0.004**
**SLL, 3 years**						
25OHD	455	-0.004 (-0.01;0.01)	0.4	501	-0.01 (-0.02;-0.002)	**0.01**
25OHD quartiles						
ref: Q1						
Q2	115	-0.22 (-0.77;0.32)	0.4	121	-0.54 (-1.02;-0.06)	**0.03**
Q3	124	-0.38 (-0.89;1.17)	0.2	123	-0.37 (-0.86;0.13)	0.15
Q4	117	-0.19 (-0.77;0.40)	0.5	129	-0.67 (-1.19;-0.15)	**0.01**

Abbreviations: 25OHD: cord S-25-hydroxyvitamin D. Q1-Q4: study specific quartiles of cord S-25-hydroxyvitamin D. SLL: sub-ischial leg length.

**Table 3:** Cord S-25-hydroxyvitamin D’s association with cephalo-caudal length:length ratio at 19 months and sitting height:height ratio at three years. The regression was adjusted for maternal pre-gestational BMI, smoking in pregnancy, maternal ethnicity, season of birth and exact child age at examination. Stratified by sex *a priori*.

	Girls			Boys		
	N	β (95% CI)	p	N	β (95% CI)	p
**CCL:L, 19 months**						
25OHD	231	-1e-05 (-1e-04;9e-05)	0.8	273	8e-05 (4e-06;1e-04)	**0.04**
25OHD quartiles						
ref: Q1						
Q2	52	-0.001 (-0.007;0.005)	0.7	67	0.002 (-0.002;0.006)	0.4
Q3	71	-0.001 (-0.007;0.005)	0.7	76	0.003 (-0.002;0.007)	0.3
Q4	65	-3e-04 (-0.006;0.006)	0.9	72	0.006 (0.001;0.01)	**0.01**
**SH:H, 3 years**						
25OHD	455	-2e-06 (-5e-05;5e-05)	0.9	501	3e-05 (-2e-05;7e-05)	0.2
25OHD quartiles						
ref: Q1						
Q2	115	0.002 (-0.003;0.003)	0.9	121	0.002 (-3e-04;0.005)	0.09
Q3	124	-1e-04 (-0.003;0.003)	0.9	123	0.002 (-4e-04;0.005)	0.09
Q4	117	-4e-04 (-0.003;0.002)	0.8	129	0.003 (3e-04;0.006)	**0.03**

Abbreviations: 25OHD: cord S-25-hydroxyvitamin D. Q1-Q4: study specific quartiles of cord S-25-hydroxyvitamin D. CCL:L-ratio: cephalo-caudal length to total length ratio. SH:H-ratio: Sitting height to total height ratio. 1e-06: 0.000001 (all such numbers).

In boys aged three years, SLL was also related to cord 25OHD, decreasing 0.1 cm for every 10 nmol/L increase in cord 25OHD, (p = 0.02), Table 2. In addition, SLL decreased with increasing cord 25OHD quartiles (linear trend, p = 0.03). A similar observation was made in SH:H increasing across quartiles of cord 25OHD (linear trend, p = 0.04), Table 3. There were no associations found between SLL, CCL:L- or SH:H-ratio and cord 25OHD in girls regardless of age, Tables 2 and 3. *Post hoc* analysis found no interactions between child sex and cord 25OHD at 19 months or three years (p = 0.2 and 0.3, respectively).

### Cord 25OHD and total length/height

Length at birth and at three months, 19 months and three years of age and their Z-scores were not associated with cord 25OHD in either boys or girls, S3 Table.

### Maternal pregnancy 25OHD levels and anthropometrics

**Fig 3:**
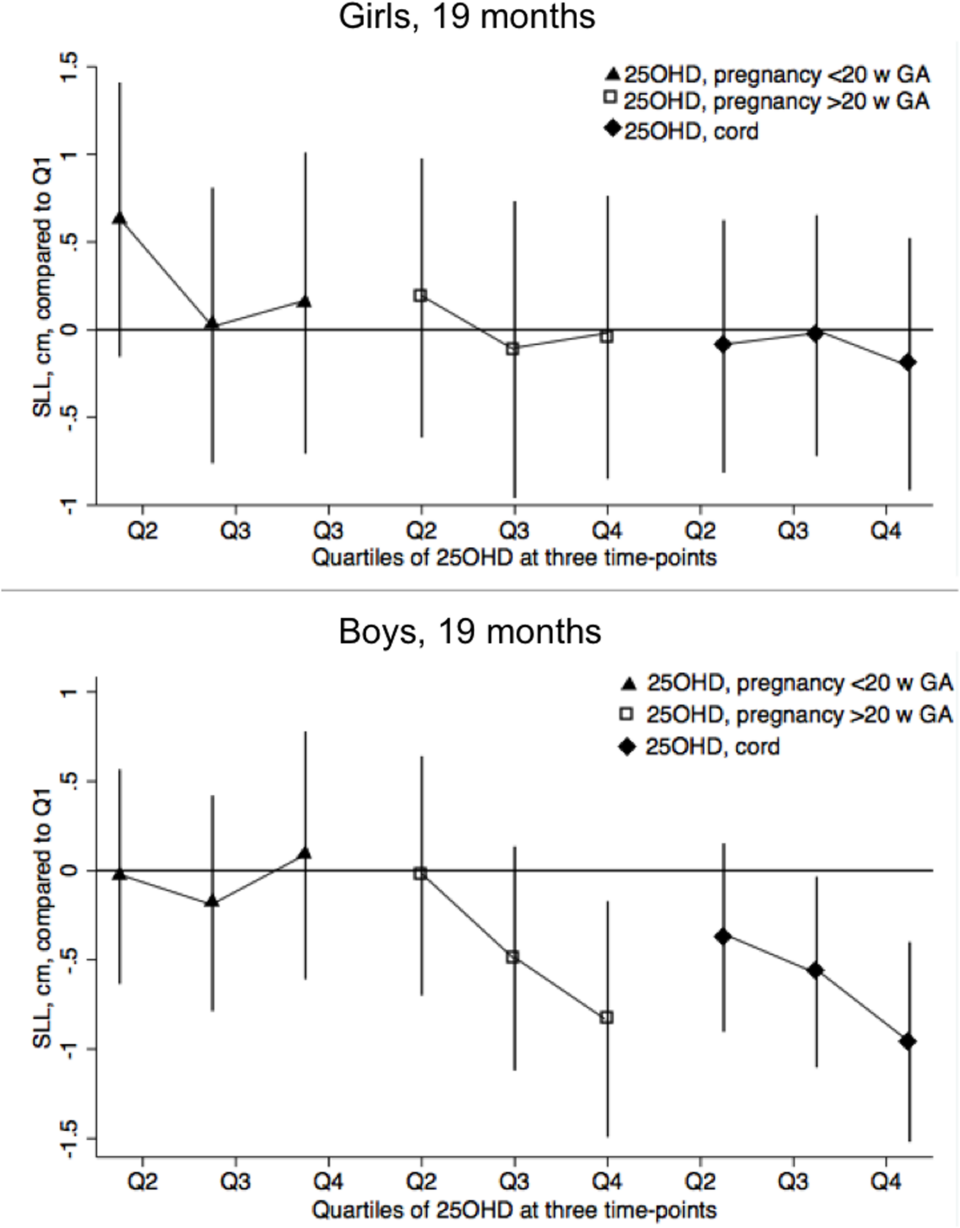
Adjusted associations between S-25-hydroxyvitamin D at different time points and leg length at 19 months of age by child sex. This is a coefficient plot of linear regression showing beta-coefficients and their 95% confidence interval. The three separate regressions illustrated model the association between S-25-hydroxyvitamin D in early pregnancy, <20 weeks gestation (triangles), in late pregnancy, >20 weeks gestation (hollow squares) and in cord serum (diamonds) and leg length of 19-months-old girls and boys. No association between early pregnancy S-25-hydroxyvitamin D and leg length seen in either sex. Late pregnancy S-25-hydroxyvitamin D shows dose-response-like quality in the relationship to leg length in boys although only Q4 reaches significance, no such pattern in girls. In boys, cord S-25-hydroxyvitamin D also shows dose-response relationship to leg length, Q3 and Q4 reaching significance, but no such pattern in girls. Abbreviations: 25OHD: S-25-hydroxyvitamin D. w: weeks. GA: gestational age.

Early pregnancy 25OHD concentrations were not associated with SLL or CCL:L-ratio at 19 months, nor to leg length or SH:H-ratio at three years in either girls or boys, Table 4 and Fig 3. In contrast, late pregnancy 25OHD was associated with SLL in 19-months-old boys with a decrease by 0.1 cm with every 10 nmol/L increase in 25OHD in adjusted analysis. The CCL:L-ratio increased accordingly with 0.001 in boys with every 10 nmol/L increase in late pregnancy 25OHD, Table 4. In 3-year old boys SLL decreased 0.,09 cm and SH:H increased 0.0006 with every 10 nmol/L increase in 25OHD. Late pregnancy 25OHD concentrations did not correlate with leg length or SH:H-ratio in girls at any age, Table 4. No *post hoc* interaction terms reached statistical significance. No consistent associations were found between early or late pregnancy 25OHD levels and total length/height Z-scores at any age in neither boys nor girls, S4 Table.

**Table 4:** Adjusted associations between S-25-hydroxyvitamin D measured in early pregnancy (<20 weeks gestation), in late pregnancy (>20 weeks gestation) and in cord serum and leg length at 19 months and three years of age and cephalo-caudal length:length ratio at 19 months of age and sitting height:height ratio at three years of age. The regression models were adjusted for maternal pre-gestational BMI, smoking in pregnancy, maternal ethnicity, season of birth and exact child age at examination. Stratified by sex *a priori*.

	Girls			Boys		
	N	β (95% CI)	p	N	β (95% CI)	p
**SLL, 19 months**						
Early pregnancy 25OHD	143	0.002(-0.01;0.02)	0.8	173	-6e-04(-0.01;0.01)	0.9
Late pregnancy 25OHD	151	-5e-05(-0.01;0.01)	0.9	167	-0.02(-0.02;-0.007)	**0.001**
Cord 25OHD	231	-0.003 (-0.02;0.009)	0.5	273	-0.02 (-0.02;-0.003)	**0.009**
**SLL, 3 years**						
Early pregnancy 25OHD	269	-0.01(-0.02;2e-05)	0.06	307	-3e-4(-0.009;0.01)	0.9
Late pregnancy 25OHD	321	-0.008(-0.01;1e-04)	0.06	342	-0.009 (-0.02;-0.002)	**0.04**
Cord 25OHD	455	-0.004 (-0.01;0.006)	0.4	501	-0.01 (-0.02;-0.002)	**0.01**
**CCL:L-ratio, 19 months**						
Early pregnancy 25OHD	143	-8e-05(-2e-04;6e-05)	0.2	173	-1e-05(-1e-04;9e-05)	0.8
Late pregnancy 25OHD	151	3e-06(-9e-05;9e-05)	0.9	167	1e-04(4e-05;2e-04)	**0.002**
Cord 25OHD	231	-1e-05(-1e-04;9e-05)	0.8	273	8e-05 (4e-06;1e-04)	**0.04**
**SH:H-ratio, 3 years**						
Early pregnancy 25OHD	269	4e-05(-2e-5;1e-4)	0.2	307	-1e-05(-6e-05;4e-05)	0.7
Late pregnancy 25OHD	321	2e-05 (-2e-05;6e-05)	0.4	342	6e-05 (2e-06;1e-04)	**0.01**
Cord 25OHD	455	-7e-06 (-4e-05;6e-05)	0.8	501	4e-05 (-2e-06;9e-05)	0.06

Abbreviations: 25OHD: S-25-hydroxyvitamin D. CCL:L-ratio: cephalo-caudal length to total length ratio. SH:H-ratio: Sitting height to total height ratio. SLL: sub-ischial leg length. 1e-6: 0.000001 (all such numbers).

## Discussion

In our prospective population-based Danish cohort of healthy children, leg shortening was not observed with lower values of pregnancy or cord 25OHD. In contrast, a novel, significant association was identified between higher late pregnancy and cord 25OHD values and shorter SLL and larger CCL:L ratios in 19-months- and 3-years-old boys, but not in girls.

### Covariates

Cord 25OHD was related with season of birth, maternal ethnicity, pre-gestational BMI, parity, maternal vitamin D supplementation and smoking in pregnancy, which is consistent with the literature [33–35]. Cord 25OHD was not associated with duration of exclusive breastfeeding or intake of infant formula after eight weeks of age and infant diet did not appear to confound the association between cord 25OHD and SLL at 19 months. Breastfed infants may be at a higher risk of rickets [36], but this did not apply to our cohort with a general high vitamin D status. In keeping with our results, longitudinal growth trajectories at age seven months were similar in breast vs. formula fed infants in an American cohort studying maternal vitamin D supplementation during lactation [37].

### Sub-ischial leg length

In a large Dutch birth cohort, comparable to the OCC e.g. regarding maternal educational level and latitude, van Eijsden *et al*. [22] found no association between early pregnancy maternal 25OHD concentration and height, leg length or relative leg length in 1208 children aged 5–6 years. This was in keeping with our findings, supporting that early pregnancy vitamin D status does not affect offspring linear growth. Our associations, linking SLL only to late pregnancy and cord 25OHD, diminished with longer time between exposure and outcome indicating that other postnatal factors may have diluted any possible effect of early life 25OHD on SLL. Van Eijsden *et al*. did not present analyses split by sex, but inverse associations between early pregnancy 25OHD and longitudinal growth in boys aged 5–6 years is probably not expected to be detectable.

As nutritional rickets is associated with short stature and reduced leg length, observing an inverse association between cord 25OHD and SLL was highly unexpected, although our population had no children with rickets and was generally healthy. We were unable to detect any direct association with SLL in the lowest 25OHD range, possibly because very low 25OHD levels were rare. Our population was therefore not suited to elucidate a potential reverse U-shaped or J-shaped association between cord 25OHD and SLL in boys.

Our dose-dependent inverse association between pregnancy and cord 25OHD and SLL at both 19 months and three years was small and hence of little clinical relevance, yet statistically significant. An underlying mechanism for causal relation remains unexplained including any theory as to why the dose-dependent pattern was only observed in males. Foetal and infant growth is a multifactorial process with major components of genetics, nutrition, and hormones, especially the insulin growth factors (IGFs) and insulin [38]. As s-IGF-I, promoting growth by proliferation of the growth plate, is inhibited by active vitamin D as demonstrated in animal and *in vitro* studies [39–43], we speculate if higher vitamin D levels may restrict infant linear growth through inhibition of IGF-I, which has higher serum concentrations in boys than in girls. Alternative explanations may be searched in the expression of genes encoding for the inactive C3-epimer vitamin D, VDR, vitamin D binding protein, etc. [44].

### Total length and height

We found no consistent associations between pregnancy or cord 25OHD and total length or height from birth to three years in either sex. Our results are supported by findings of van Eijsden *et al*. [22] in a Dutch cohort. Also Ong *et al*. [45] observed no association between maternal 25OHD sampled at 26–28 weeks gestation and offspring length Z-scores at 18 months in 596 children of mixed ethnicity from Singapore with a lower rate of vitamin D insufficiency (25%) and deficiency (6%) compared to our mothers (40% insufficient and 17% deficient). In contrast, Eckhardt *et al*. [21] observed smaller length for age Z-scores in children whose mothers had pregnancy 25OHD levels (26 weeks’ gestation) < 30 nmol/L with consistent results across the first year of life. The smaller length for age Z-scores in Eckhardt’s <30 nmol/L group may have been undetectable in our sample as we had only twelve late pregnancy 25OHD observations below this cut-off. Taken together, pregnancy and cord 25OHD had no clinically important associations with linear growth in our generally healthy population of infants.

### Strengths and limitations

Strengths of the present study include its large sample size, prospective design, low dropout rate, longitudinal measurements of serum 25OHD_2+3_, by use of the gold standard LC-MS/MS method, and the inclusion of both early pregnancy, late pregnancy and cord 25OHD for the detection of time windows of effect. *Post hoc* power calculations further indicated that the study had sufficient power to identify true differences in SLL. Nonetheless, our findings warrant replication in other child cohorts and follow up to older child ages.

Our study was limited by the observational design disallowing causal conclusions. While our design favoured conclusions on the primary association (cord s-25OHD and SLL at 1½ years), chance findings could not be ruled out in the secondary associations due to multiple testing. Furthermore, we cannot exclude the possibility of residual confounding caused by unmeasured or poorly measured factors. Information bias may be expected using questionnaires; however potential misclassification was expected to be non-differential between parents having children of each sex. Data were missing, especially in cephalo-caudal length at 19 months. Most of the missing values were, however, caused by a temporary non-inclusion of cephalo-caudal length measurements for 12 months halfway through the three-year study period, hence missing data is expected to be random. Our participants were comparable with the local Danish background population, except for fewer participants of non-Danish origin, fewer smokers during pregnancy and more with higher education [27].

### Conclusion

In a healthy child population with very few having vitamin D deficiency, lower 25OHD did not associate with reduced leg length. In contrast, we identified a novel but small, significant association between increasing late pregnancy and cord 25OHD and shorter leg length in 19-months and 3-years-old-boys, but not in girls. Early 25OHD did not associate to total body length. In conclusion, early life vitamin D status had no clinically important associations with linear growth in a generally healthy population of infants.

## Supporting information

S1 Table Descriptive statistics of secondary outcomes. Table of the descriptive statistics of wrist circumference to antebrachium circumference ratio, length for age Z-scores at birth, three months and 19 months of age and height for age Z-scores at three years. Z-scores were calculated based on Danish references.
(DOCX)

S2 Table Crude associations between cord 25-hydroxyvitamin D and all outcomes of linear growth. Table of the crude linear regressions modelling cord S-25-hydroxyvitamin D (25OHD) and sub-ischial leg length (SLL) at 19 months, as well as cord 25OHD and SLL at three years, birth length, birth length for gestational age Z-score (BLZ), length at three months, length for age (LAZ) at three months, length at 19 months, LAZ at 19 months, height at three years and height for age (HAZ) at three years. The table shows β coefficient estimates including their 95% confidence interval (CI) from the crude linear regressions.
(DOCX)

S3 Table Adjusted associations between cord S-25-hydroxyvitamin D and secondary outcomes of linear growth including birth length, birth length for gestational age Z-score (BLZ), length at three months, length for age (LAZ) at three months, length at 19 months, LAZ at 19 months, height at three years and height for age (HAZ) at three years. The table includes linear regression beta-coefficient estimates and 95% confidence intervals. All regression models were adjusted for maternal pre-gestational BMI, smoking in pregnancy, maternal ethnicity, season of birth and exact child age at examination. Stratified by sex *a priori*. Includes outcomes: birth length, birth length for gestational age Z-score (BLZ), length at three months, length for age (LAZ) at three months, length at 19 months, LAZ at 19 months, height at three years and height for age (HAZ) at three years.
(DOCX)

S4 Table The association between S-25-hydroxyvitamin D and length/height for age adjusted Z-scores. S-25-hydroxyvitamin D was measured in early pregnancy (<20 weeks gestation), in late pregnancy (>20 weeks gestation) and in cord serum. No consistent significant associations seen. Linear regression beta-coefficient estimates and 95% confidence intervals. All regression models were adjusted for maternal pre-gestational BMI, smoking in pregnancy, maternal ethnicity, season of birth and exact child age at examination. Stratified by sex *a priori*.
(DOCX)
