# Supplementary material for: A novel inverse association between cord 25-hydroxyvitamin D and leg length in boys up to three years. An Odense Child Cohort study
Source: PLoS One. 2018 Jun 11;13(6):e0198724. doi: 10.1371/journal.pone.0198724 (PMC5995352; doi:10.1371/journal.pone.0198724)
Supplement: S1 Table — Table of the descriptive statistics of wrist circumference to antebrachium circumference ratio, length for age Z-scores at birth, three months and 19 months of age and height for age Z-scores at three years. Z-scores were calculated based on Danish references. (DOCX) [file pone.0198724.s001.docx]

**S1** **Table. Descriptive statistics of secondary outcomes.** Table of the descriptive statistics of length for age Z-scores at birth, three months and 19 months of age and height for age Z-scores at three years. Z-scores were calculated based on Danish references. All ages provided in months.

|  |  | Girls | | | Boys | | |
| --- | --- | --- | --- | --- | --- | --- | --- |
|  | Mean (SD) age | N | Mean (SD) | Z-scores  mean (SD) | N | Mean (SD) | Z-scores  mean (SD) |
| **Birth length, cm** | 9.2 (0.3)* | 976 | 51.57 (2.3) | 0.005 (1.0) | 1097 | 52.37 (2.3) | 0.002 (1.0) |
| **Length, cm** | 2.8 (0.9) | 860 | 62.20 (2.6) | 0.08 (0.9) | 958 | 64.1 (2.71) | 0.18 (0.9) |
| **Length, cm** | 19.1 (0.9) | 599 | 83.20 (3.0) | 0.33 (1.0) | 719 | 84.82 (2.9) | 0.27 (0.9) |
| **Height, cm** | 36.6 (0.8) | 491 | 96.08 (3.60) | 0.07 (1.0) | 554 | 97.57 (3.5) | 0.09 (0.9) |

*SD according to gestational age in weeks.
