# Supplementary material for: A novel inverse association between cord 25-hydroxyvitamin D and leg length in boys up to three years. An Odense Child Cohort study
Source: PLoS One. 2018 Jun 11;13(6):e0198724. doi: 10.1371/journal.pone.0198724 (PMC5995352; doi:10.1371/journal.pone.0198724)
Supplement: S2 Table — Table of the crude linear regressions modelling cord S-25-hydroxyvitamin D (25OHD) and sub-ischial leg length (SLL) at 19 months, as well as cord 25OHD and SLL at three years, birth length, birth length for gestational age Z-score (BLZ), length at three months, length for age (LAZ) at three months, length at 19 months, LAZ at 19 months, height at three years and height for age (HAZ) at three years. The table shows β coefficient estimates including their 95% confidence interval (CI) from the crude linear regressions. (DOCX) [file pone.0198724.s002.docx]

**S2 Table. Crude associations between cord 25-hydroxyvitamin D and all outcomes of linear growth**. Table of the crude linear regressions modelling cord S-25-hydroxyvitamin D (25OHD) and sub-ischial leg length (SLL) at 19 months, as well as cord 25OHD and SLL at three years, birth length, birth length for gestational age Z-score (BLZ), length at three months, length for age (LAZ) at three months, length at 19 months, LAZ at 19 months, height at three years and height for age (HAZ) at three years. The table shows β coefficient estimates including their 95% confidence interval (CI) from the crude linear regressions.

|  | Total | | Girls | | Boys | |
| --- | --- | --- | --- | --- | --- | --- |
|  | N | β (95% CI) | N | β (95% CI) | N | β (95% CI) |
| SLL, 19 months | 504 | -0.004 (-0.01;0.003) | 231 | -3e-04 (-0.01;0.01) | 273 | -0.006 (-0.01;0.002) |
| SLL, 3 years | 956 | -0.006* (-0.01;-2e-04) | 455 | -0.004 (-0.01;0.005) | 501 | -0.008* (-0.02;-2e-04) |
| CCL:L-ratio, 19 months | 504 | 1e-05 (-4e-05;6e-05) | 231 | -2e-05 (-1e-04;7e-05) | 273 | 4e-05 (-3e-05;1e-04) |
| SH:H-ratio, 3 years | 956 | 9e-06 (-2e-05;4e-05) | 455 | -6e-06 (-5e-05;4e-05) | 501 | 3e-05 (-1e-05;6e-06) |
| Birth length | 2073 | -9e-04 (-0.006;0.004) | 976 | -0.001 (-0.008;0.005) | 1097 | -5e-04 (-0.007;0.006) |
| BLZ | 2070 | 1e-05 (-0.002;0.002) | 975 | 4e-04 (-0.003;0.003) | 1095 | -3e-04 (-0.003;0.002) |
| Length, 3 months | 1818 | -0.006* (-0.12;-6e-05) | 860 | -0.003 (-0.01;0.006) | 958 | -0.008* (-0.02;-5e-04) |
| LAZ, 3 months | 1817 | -0.001 (-0.003;9e-04) | 860 | -0.001 (-0.004;0.002) | 957 | -0.001 (-0.004;0.002) |
| Length, 19 months | 1318 | -0.006 (-0.01;0.001) | 599 | -0.008 (-0.02;0.003) | 719 | -0.003 (-0.01;0.007) |
| LAZ, 19 months | 1317 | -0.002 (-0.005;6e-05) | 598 | -0.003 (-0.007;9e04) | 719 | -0.002 (-0.006;0.001) |
| Length, 3 years | 1045 | -0.01* (-0.02;-0.0003) | 491 | -0.009 (-0.02;0.006) | 554 | -0.01 (-0.02;0.003) |
| HAZ, 3 years | 1045 | -0.002 (-0.005;0.0006) | 491 | -0.002 (-0.006;0.002) | 554 | -0.002 (-0.006;0.001) |

* p-value <0.05. SLL = sub-ischial leg length. CCL:L-ratio= cranio-caudal length to length ratio. SH:H-ratio = sitting height to total height ratio. BLZ = birth length for gestational age Z-score. LAZ = length for age Z-score. HAZ = height for age Z-score. 1e-06 = 0.000001 (all such numbers).
