# Supplementary material for: A novel inverse association between cord 25-hydroxyvitamin D and leg length in boys up to three years. An Odense Child Cohort study
Source: PLoS One. 2018 Jun 11;13(6):e0198724. doi: 10.1371/journal.pone.0198724 (PMC5995352; doi:10.1371/journal.pone.0198724)
Supplement: S3 Table — The table includes linear regression beta-coefficient estimates and 95% confidence intervals. All regression models were adjusted for maternal pre-gestational BMI, smoking in pregnancy, maternal ethnicity, season of birth and exact child age at examination. Stratified by sex a priori. Includes outcomes: birth length, birth length for gestational age Z-score (BLZ), length at three months, length for age (LAZ) at three months, length at 19 months, LAZ at 19 months, height at three years and height for age (HAZ) at three years. (DOCX) [file pone.0198724.s003.docx]

**S3 Table. Adjusted associations between cord S-25hydroxyvitamin D and secondary outcomes of linear growth including birth length, birth length for gestational age Z-score (BLZ), length at three months, length for age (LAZ) at three months, length at 19 months, LAZ at 19 months, height at three years and height for age (HAZ) at three years.** The table includes linear regression beta-coefficient estimates and 95 % confidence intervals. All regression models were adjusted for maternal pre-gestational BMI, smoking in pregnancy, maternal ethnicity, season of birth and exact child age at examination. Stratified by sex *a priori*.

|  | Girls | | Boys | | |
| --- | --- | --- | --- | --- | --- |
|  | N | β (95% CI) | N | β (95% CI) | |
| **Birth Length** |  |  |  |  | |
| 25OHD | 974 | 0.002(-0.004;0.008) | 1095 | -0.009(-0.007;0.005) | |
| 25OHD quartiles |  |  |  |  | |
| Q2 |  | 0.15 (-0.19;0.49) |  | -0.19 (-0.52;0.14) | |
| Q3 |  | 0.05 (-0.29;0.40) |  | -0.19 (-0.54;0.15) | |
| Q4 |  | 0.07 (-0.30;0.43) |  | -0.10 (-0.46;-0.26) | |
| **BLZ** |  |  |  |  | |
| 25OHD | 974 | 0.001(-0.002;0.004) | 1095 | 3e-05(-0.003;0.003) | |
| 25OHD quartiles |  |  |  |  | |
| Q2 |  | 0.07 (-0.09;0.23) |  | -0.08 (-0.23;0.06) | |
| Q3 |  | 0.03 (-0.13;0.18) |  | -0.09 (-0.24;0.07) | |
| Q4 |  | 0.03 (-0.14;0.20) |  | -0.05 (-0.22;0.11) | |
| **Length, 3 months** |  |  |  |  | |
| 25OHD | 860 | -7e-04 (-0.008;0.007) | 958 | -0.002(-0.01;0.004) | |
| 25OHD quartiles |  |  |  |  | |
| Q2 |  | 0.04 (-0.40;0.47) |  | -0.23 (-0.64;0.18) | |
| Q3 |  | -0.06 (-0.49;0.38) |  | -0.28 (-0.71;0.15) | |
| Q4 |  | 0.02 (-0.44;0.48) |  | -0.19 (-0.63;0.26) | |
| **LAZ, 3 months** |  |  |  |  | |
| 25OHD | 860 | -3e-04 (-0.004;0.003) | 958 | -1e-04(-0.003;0.003) | |
| 25OHD quartiles |  |  |  |  | |
| Q2 |  | 0.04 (-0.16;0.24) |  | -0.10 (-0.29;0.08) | |
| Q3 |  | -0.03 (-0.23;0.17) |  | -0.07 (-0.26;0.12) | |
| Q4 |  | 0.02 (-0.19;0.23) |  | -0.05 (-0.26;0.15) | |
| **Length, 19 months** |  |  |  | |  |
| 25OHD | 595 | -0.01 (-0.02;0.002) | 715 | -0.007(-0.02;0.004) | |
| 25OHD quartiles |  |  |  |  | |
| Q2 |  | -0.49 (-1.17;0.20) |  | -0.13 (-0.72;0.45) | |
| Q3 |  | -0.61 (-1.30;0.08) |  | -0.20 (-0.81;0.41) | |
| Q4 |  | -0.54 (-1.26;0.19) |  | -0.38 (-1.02;0.27) | |
| **LAZ, 19 months** |  |  |  |  | |
| 25OHD | 598 | -0.003 (-0.08;9e-04) | 719 | -0.003(-0.06;0.001) | |
| 25OHD quartiles |  |  |  |  | |
| Q2 |  | -0.20 (-0.44;0.05) |  | -0.06 (-0.27;0.14) | |
| Q3 |  | -0.22 (-0.47;0.02) |  | -0.08 (-0.30;0.14) | |
| Q4 |  | -0.20 (-0.45;0.06) |  | -0.14 (-0.37;0.08) | |
| **Height, 3 years** |  |  |  |  | |
| 25OHD | 487 | -0.009 (-0.03;0.008) | 552 | -0.01(-0.03;0.003) | |
| 25OHD quartiles |  |  |  |  | |
| Q2 |  | -0.43 (-1.39;0.53) |  | -0.63 (-1.45;0.19) | |
| Q3 |  | -0.90 (-1.84;0.05) |  | -0.28 (-1.13;0.57) | |
| Q4 |  | -0.38 (-1.40;0.63) |  | -0.78 (-1.67;0.11) | |
| **HAZ, 3 years** |  |  |  |  | |
| 25OHD | 491 | -0,002 (-0.007;0.002) | 554 | -0.003 (-0.008;8e-04) | |
| 25OHD quartiles |  |  |  |  | |
| Q2 |  | -0.12 (-0.40;0.15) |  | -0.18 (-0.40;0.05) | |
| Q3 |  | -0.26 (-0.53;0.004) |  | -0.08 (-0.32;0.16) | |
| Q4 |  | -0.12 (-0.40;0.18) |  | -0.22 (-0.47;0.03) | |

*P-value < 0.05. 25OHD = cord S-25hydroxyvitamin D. Q1-Q4 = study specific quartiles of cord S-25hydroxyvitamin D (ref: Q1). WC:AC-ratio = wrist circumference to antebrachium circumference ratio. LAZ = length for age adjusted Z-scores. HAZ = height for age adjusted Z-scores. 1e-06 = 0.000001 (all such numbers)
