# Supplementary material for: A novel inverse association between cord 25-hydroxyvitamin D and leg length in boys up to three years. An Odense Child Cohort study
Source: PLoS One. 2018 Jun 11;13(6):e0198724. doi: 10.1371/journal.pone.0198724 (PMC5995352; doi:10.1371/journal.pone.0198724)
Supplement: S4 Table — No consistent significant associations seen. Linear regression beta-coefficient estimates and 95% confidence intervals. All regression models were adjusted for maternal pre-gestational BMI, smoking in pregnancy, maternal ethnicity, season of birth and exact child age at examination. Stratified by sex a priori. (DOCX) [file pone.0198724.s004.docx]

**S4 Table. The association between S-25-hydroxyvitamin D and length/height for age adjusted Z-scores. S-25-hydroxyvitamin D was measured in early pregnancy (<20 weeks gestation), in late pregnancy (>20 weeks gestation) and in cord serum.** No consistent significant associations seen. Linear regression beta-coefficient estimates and 95% confidence intervals. All regression models were adjusted for maternal pre-gestational BMI, smoking in pregnancy, maternal ethnicity, season of birth and exact child age at examination. Stratified by sex *a priori*.

|  |  | Girls |  | Boys |
| --- | --- | --- | --- | --- |
|  | N | β (95% CI) | N | β (95% CI) |
| **BLZ** |  |  |  |  |
| Early pregnancy  S-25OHD | 529 | -0.004 (-0.008;2e-04) | 647 | -8e-04 (-0.004;0.003) |
| Late pregnancy  S-25OHD | 638 | 0.001 (-0.004;0.001) | 718 | -0.003 (-0.005;0.002) |
| Cord S-25OHD | 974 | 0.001 (-0.002;0.004) | 1095 | 3e-05 (-0.003;0.003) |
| **LAZ, 3 months** |  |  |  |  |
| Early pregnancy  S-25OHD | 513 | -0.003 (-0.008;0.001) | 577 | 8e-04 (-0.003;0.005) |
| Late pregnancy  S-25OHD | 588 | -0.002 (-0.005;0.001) | 651 | -0.002 (-0.005;0.002) |
| Cord S-25OHD | 860 | -3e-04 (-0.004;0.003) | 958 | -1e-04 (-0.003;0.03) |
| **LAZ, 19 months** |  |  |  |  |
| Early pregnancy  S-25OHD | 360 | -0.006 (-0.01;0.001) | 438 | 8e-04 (-0.003;0.005) |
| Late pregnancy  S-25OHD | 410 | -0.003 (-0.007;8e-04) | 493 | -0.002 (-0.006;0.001) |
| Cord S-25OHD | 598 | -0.003 (-0.008;9e-04) | 719 | -0.002 (-0.006;0.001) |
| **HAZ, 3 years** |  |  |  |  |
| Early pregnancy  S-25OHD | 290 | -0.005 (-0.01;4e-04) | 335 | -0.001 (-0.006;0.004) |
| Late pregnancy  S-25OHD | 344 | -0.004 (-0.008;4e-04) | 378 | -0.002 (-0.006;0.003) |
| Cord S-25OHD | 491 | -0,003 (-0.007;0.002) | 554 | -0.003 (-0.008;8e-04) |

*P-value < 0.05 S-25OHD = S-25hydroxyvitamin D. Q1-Q4 = study specific quartiles of cord S-25-hydroxyvitamin D. BLZ = birth length for gestational age adjusted Z-score. LAZ = length for age adjusted Z-scores. HAZ = height for age adjusted Z-scores. 1e-06 = 0.000001 (all such numbers)
